# Asymptomatic Carotid Stenosis Is Associated With Circadian and Other Variability in Embolus Detection

**DOI:** 10.3389/fneur.2019.00322

**Published:** 2019-04-16

**Authors:** Anne L. Abbott, Julia Merican, Dora C. Pearce, Ana Juric, Christopher Worsnop, Emma Foster, Brian Chambers

**Affiliations:** ^1^Central Clinical School, Monash University, Melbourne, VIC, Australia; ^2^Neurology Network, Knox Private Hospital, Wantirna, VIC, Australia; ^3^Neurology and Neurosurgery Centre, Princecourt Medical Centre, Kuala Lumpur, Malaysia; ^4^Faculty of Medicine, Dentistry and Health Sciences, University of Melbourne, Melbourne, VIC, Australia; ^5^School of Science, Engineering and Information Technology, Federation University, Ballarart, VIC, Australia; ^6^Independent Researcher, Melbourne, VIC, Australia; ^7^Department of Respiratory and Sleep Medicine, Austin Health, Melbourne, VIC, Australia; ^8^Neurology Department, Alfred Health, Melbourne, VIC, Australia; ^9^Neurology Department, The Royal Melbourne Hospital, Melbourne, VIC, Australia; ^10^Department of Neurology, Austin Health, Melbourne, VIC, Australia; ^11^Faculty of Medicine, University of Melbourne, Melbourne, VIC, Australia

**Keywords:** carotid stenosis, circadian, embolism, transcranial Doppler, sleep apnea, stroke

## Abstract

**Background and Purpose:** Variability in transcranial Doppler (TCD) detection of embolic signals (ES) is important for risk stratification. We tested the effect of time of day on ES associated with 60–99% asymptomatic carotid stenosis.

**Materials and Methods:** Subjects were from the Asymptomatic Carotid Stenosis Embolus Detection (ASED) Study such that half were previously ES-positive and half ES-negative with 6-monthly 60-min TCD monitoring. All underwent bilateral TCD monitoring for two 12-h sessions separated by 24 h. ES detection rates were calculated using 6 and 4-h intervals from midnight and effective TCD monitoring time.

**Results:** Ten subjects (8 male, mean age 79.5 years) were monitored. Over 24 h, 5/10 study arteries with 60–99% asymptomatic carotid stenosis were ES-positive (range 1–28 ES/artery, 56 total ES from 177.9 total effective monitoring hours). The remaining five study arteries and all eight successfully monitored contralateral arteries were ES-negative. Using 6-h intervals the mean ES detection rate peaked at 0600-midday (0.64/h) and was lowest 1800-midnight (0.09/h) with an incidence rate ratio of 7.26 (95% CI 2.52–28.64, *P* ≤ 0.001). Using 4-h intervals the mean ES detection rate peaked at 0800-midday (0.64/h) and was lowest midnight-0400 (0.12/h) with an incidence rate ratio of 5.51 (95% CI 1.78–22.67, *P* = 0.001).

**Conclusions:** Embolism associated with asymptomatic carotid stenosis shows circadian variation with highest rates 4–6 h before midday. This corresponds with peak circadian incidence of stroke and other vascular complications. These and ASED Study results show that monitoring frequency, duration, and time of day are important in ES detection.

## Introduction

Transcranial Doppler (TCD) detected microembolism in the ipsilateral middle cerebral artery (MCA) may help stratify the risk of stroke and other arterial disease complications in persons with advanced (≥60%) asymptomatic carotid stenosis. If so, this technique could lead to improved prevention. However, TCD embolic signal (ES) detection is particularly labour intensive in asymptomatic individuals due to relatively low ES detection rates. Approximately 10% of individuals have at least 1–2 ES detected within 1–2 h of TCD monitoring, with median hourly ES rates among ES-positive arteries of 1–2 (1–3). In the Asymptomatic Stenosis Embolus Detection (ASED) Study, on average one ES was detected for every 6.25 h of TCD monitoring (1). The efficiency of TCD monitoring to identify microembolism could be improved if factors influencing shedding rates, including time of day, were better identified.

It is unknown if time of day has a significant influence on detecting microembolism associated with asymptomatic carotid stenosis. However, preliminary data from a cohort of seven symptomatic persons with different potential embolic sources and TCD monitored for 24 h suggests that there may be an effect (4). Further, higher rates of ES are detected in the first 3 h following carotid endarterectomy (CEA) performed in the morning compared to the afternoon (5). There is also a well-documented late-morning peak in incidence of symptomatic arterial and venous disease complications, including ischaemic and haemorrhagic stroke (6), transient ischaemic attack (6), subarachnoid haemorrhage (7), unstable angina and myocardial infarction (8, 9) coronary artery bare metal stent thrombosis (10) sudden cardiac death (8) acute aortic dissection (11), and pulmonary embolism (12, 13) Factors implicated in the pathogenesis of these vascular catastrophes also show a morning peak in activity, usually in the 6 h before midday. These include blood pressure and heart rate (14, 15) blood adrenaline levels (16) platelet aggregation (17, 18) blood fibrinolytic activity (19), and haematocrit (20). The aim of this study was to check for circadian and other variation in TCD detected microembolism associated with advanced asymptomatic carotid stenosis in order to improve risk stratification.

## Materials and Methods

### Subject Selection

All subjects for 24-h TCD were selected from the Asymptomatic Carotid Stenosis Embolus Detection (ASED) Study which was designed to determine the value of TCD embolus detection in stratifying the risk of ipsilateral stroke or transient ischaemic attack (TIA) in relation to advanced asymptomatic carotid stenosis (1). In brief, in this multicentre study all subjects underwent 6-monthly clinical assessment (including blood pressure measurement), bilateral carotid duplex and 60-m TCD monitoring of the MCA ipsilateral to asymptomatic duplex-determined 60–99% carotid stenosis during office hours. ASED Study subjects had no previous clinically defined stroke or TIA involving the ipsilateral brain or retina (1). However, past contralateral or vertebrobasilar stroke or TIA and clinically silent imaging evidence of brain ischaemia in any vascular territory were allowed (1). During ASED Study follow-up details about the presence of arterial disease risk factors and outcome events were reported back to the general practitioners. Study investigators did not usually intervene directly with respect to patient management unless there were very compelling reasons, such as the new discovery of stroke or TIA or atrial fibrillation.

ASED Study criteria for 60–99% carotid stenosis included a proximal ICA peak systolic velocity (PSV) of ≥150 cm/s. Other criteria varied according to study centre. The great majority (≥90%) of subjects were recruited at the Austin Hospital (principal study site) which used criteria after Zweibel and Bluth (21, 22), see Supplementary Table 1. At the time of the ASED study, audits of carotid duplex stenosis grading in comparison with digital subtraction angiography and NASCET criteria were performed regularly at the Austin Hospital. The sensitivity and specificity of detecting of carotid stenosis >70% using carotid duplex was found to be 88 and 96%, respectively, based on a sample of 81 patients in the 4 years after mid-1997, similar to previous audit results (23). Subjects from the secondary sites (Box Hill and John Hunter Hospitals) were recruited using criteria from the Australasian Society for Ultrasound Medicine (1,998 conference presentation, (24), Supplementary Table 2). Sonographers were asked to grade stenosis according to decile categories from 40–49% to 80–99% or as <40% or as occluded. If the sonographer's result varied from these ASED study ranges (as sometimes occurred at secondary sites), the point estimate or the midpoint of the reported stenosis range was used to categorise stenosis severity.

Subjects without an adequate ipsilateral temporal bone insonation window were excluded from the ASED Study, as were those with limited life expectancy and any known non-study carotid arterial disorders associated with cerebral embolism (prosthetic heart valves, chronic congestive cardiac failure, electrocardiographically documented chronic/recurrent atrial fibrillation and known mobile aortic arch atheroma were the reasons for exclusion during screening). ASED Study subjects were asked to have 24-h TCD according to who was perceived as most able and willing to tolerate prolonged monitoring. In addition, to allow the best chance of representing both “embolising” and “non-embolising” arteries, half the subjects had been previously ES-positive (with ≥1 ES) and half always ES-negative during prior routine ASED Study 6-monthly, 60-min TCD monitoring (1). At the time of 24-h monitoring in late 2000-early 2001, 201 of the total 202 ASED study patients had had ≥1 h of TCD monitoring and all were considered for the 24-h studies.

### Arterial Disease Risk Factor Definition

Hypertension, diabetes and hypercholesterolaemia were diagnosed using history or direct measurement (blood pressure of ≥160/90, fasting blood glucose of ≥7.5 mmol/l and fasting total serum cholesterol of ≥5.5 mmol/l). Current smoking was defined as any tobacco smoking in the preceding 3 months after habitual intake for ≥12 months. A past smoker had abstained for ≥3 months. Ischaemic heart disease indicated a history of angina pectoris, myocardial infarction (using clinical features, electrocardiographs, and cardiac enzymes) or ischaemic cardiac failure. Peripheral vascular disease indicated a history of lower limb arterial insufficiency (claudication, gangrene, or arterial disease procedures), aortic aneurysm, or known atherosclerotic renal artery stenosis (1).

#### Twenty Four-Hour TCD Monitoring

Bilateral MCA TCD monitoring was conducted during two 12-h sessions (0900–2100 and 2100–0900) each separated by 24 h. To avoid bias from overly representing results from individuals, in the presence of bilateral 60–99% asymptomatic ICA stenosis, the most severely stenotic ICA was considered a “study artery” and the contralateral ICA was considered a “control.” If bilateral monitoring (for equipment or subject reasons) was not possible, the MCA ipsilateral to the “study ICA artery” was monitored.

ES monitoring studies were performed during the Australian spring/summer months (October 2000–January 2001). Subjects spent most of the monitoring time in a comfortable recliner chair. TCD cabling allowed short walks within the room. Talking by subjects during monitoring was discouraged. Short monitoring breaks were permitted, including approximately 30 min for lunch, when monitoring ceased and the headset was removed. For logistical and exploratory research reasons, all overnight TCD monitoring was performed during a simultaneous formal sleep study to detect evidence of sleep disordered breathing, particularly obstructive sleep apnea (OSA, see below).

TCD monitoring was performed using a Multidop T2 (DWL Elekronische System GmbH) and a Spencer headset for probe fixation. MCA insonation, using the temporal acoustic window, was performed at a depth of 50–60 mm using a 2 MHz pulsed Doppler transducer. The sample volume (generally 8–15 mm), emitted power (<100 mW) and gain were minimised to optimise the ES to background signal relationship. An investigator (JM) trained in MCA insonation sat with all subjects during TCD monitoring to continually optimise the signal. An eight-channel Sony PCM-800 DAT recorder was used for all monitoring except when circumstances allowed only unilateral monitoring, in which case a two-channel Sony DAT recorder was employed.

### Embolic Signal Analysis

All ES analysis in the ASED and 24-h study was performed “off-line” using the EME-Nicolet Pioneer TC 2020 in two stages by trained observers blinded to all subject information, including degree of carotid stenosis and ES detection results. Initially an observer screened the whole recording noting the timing of possible ES and with instruction to be over- rather than under-inclusive (to maximise sensitivity). Then two observers (AA and SM or AJ) validated these results and maximised specificity by together inspecting each possible ES using Consensus Committee criteria (25). Typical ES, as agreed by both validators, had a crisp sound, were unidirectional within the Doppler velocity spectrum and had an intensity of ≥6 dB above the background flow signal as determined manually using an average of several measurements using the replay device colour-intensity scale. Averages of 5.5–6 dB were rounded to 6 dB. Typical ES were differentiated from artefacts (most often due to TCD probe movement or contact, superficial temporal artery pulsation and the subject talking). To help standardisation, all ES analysis was performed at one center (Austin Health) and one investigator (AA) was always involved in validation. The yield of detecting ES from 24-h monitoring was compared to repeated 60-min TCD monitoring the same day or up to 6-monthly as occurred during the ASED Study (1).

### Reliability of Our Embolic Signal Detection Method

Our ES detection methods, including for the choice of 6 dB ES detection threshold, were derived from previous experiments to test equipment and personnel factors (23, 26, 27). In addition, before any involvement in ES detection, every investigator had to obtain a sensitivity of ≥90% in detecting typical ES on an internationally utilised reference digital audio tape (DAT) which contained ES from patients with symptomatic carotid stenosis and common artefacts, such as those produced by probe contact, talking, or coughing (27). Later, new personnel also had to achieve a sensitivity of at ≥90% in detecting ES in association with ≥1 ASED Study subjects and ≥1 subject known to be ES-positive after CEA at Austin Health (28).

As a measure of agreement in ES analysis, we compared the results of sixty 30–60 min TCD monitoring studies from 46 different ASED study arteries which were inadvertently screened and validated at least twice while still blinded to subject identity (including previous ES results). Each replicate was matched to the original ES result on file. This gave rise to 58 concordant pairs (both ES-negative in 57 cases and both ES-positive in 1 case) and 2 discordant pairs (different with respect detecting ≥1 ES). This demonstrates a 97% concordance. The exact symmetry test (exact McNemar *P* = 1.000) further indicated negligible change in emboli detection and hence repeatability of our ES analysis technique (https://www.stata.com/manuals13/rsymmetry.pdf). The 2 cases with different results illustrated the occasional problem of varied interpretation, usually in cases border-line with respect to accepted ES criteria and/or signal intensity (25). Such uncertainty is not necessarily clinically significant.

### Sleep Disordered Breathing Assessment

Using history and examination all subjects were assessed prior to 24-h TCD monitoring (author AA) and given a subjective pre-test probability (0–100%) of having OSA. This probability was categorised as low (<30%), intermediate (31–69%), or high (70–100% or having a past history of sleep-study confirmed OSA). Subjects were asked about a previous diagnosis of OSA, previous sleep studies, snoring, choking during sleep, sleep quality, and day-time somnolence. All over-night TCD monitoring and coincident sleep studies were performed without ventilatory assistance (including continuous positive airway pressure, CPAP). Sleep study results were interpreted by author CW using the Chicago 1999 criteria and blinded to ES results (29).

### Statistical Analysis

Mean hourly ES detection rates were calculated according to four 6-h intervals and six 4-h intervals from midnight, taking into account monitoring breaks and signal recording adequacy (“effective” monitoring). Incidence rate ratios of peak compared to lowest ES detection rates were calculated and exact *P*-values and 95% confidence intervals (CI) reported (Stata/SE 14.2 for Windows, 2017; StataCorp LLC, 4905 Lakeway Drive, College Station, TX 77845 USA). ES detection yield using 24-h TCD monitoring was compared to that using 60-min monitoring in the ASED Study (1).

### Ethical Considerations

This study was granted Austin Health Ethics Committee approval and all participants gave written informed consent.

## Results

### Demographics and General Arterial Disease Risk Factors

Ten subjects (eight male) underwent 24-h of bilateral MCA TCD monitoring for ES detection. Nine subjects were recruited into the ASED Study from the Austin Hospital and one from Box Hill Hospital (both in Melbourne). Tables 1, 2 show the degree of carotid stenosis bilaterally, demographics, other risk factors, and medications taken in relation to the 24-h study. Four subjects reported past non-study ICA territory stroke or TIA. These events were confirmed by the medical records for subjects 2, 6 (who had CEA after a left cerebral hemispheric TIA), and 7 (who had a right cerebral hemispheric stroke with left hemiparesis and brain imaging evidence of corresponding lacunar infarction). All 10 subjects were neurologically normal at the time of 24-h TCD. Study ICA plaque morphology (according to the closest carotid duplex study performed before 24-h TCD monitoring) was characterised as calcified in subjects 1 and 10 (obscuring other plaque morphology), as irregular in subjects 4–9, as “heterogeneous” or “mixed” (echolucent and echogenic) with or without some calcification in subjects 2, 4, 8, and 9 and as “homogeneous” (uniformly echogenic) in subjects 3, 6, and 7.

**Table 1:** Degree of ICA stenosis at the most recent duplex scan prior to 24-h TCD monitoring ^†^.

**Patient/artery number**	**Study ICA side**	**Degree of study ICA stenosis (%)**	**Study ICA PSV**	**Study ICA EDV**	**Study ICA stenosis summary**	**Contralateral ICA stenosis degree**	**Contralateral ICA PSV**	**Contralateral ICA EDV**
1	Right	60–70	205	48	Stable Since December 1998	0	54	16
2	Left	50–69^*^	177	54	Stable since March 2000	<20	56	17
3	Left	60–70	179	34	Stable Since October 1999	Occluded	0	0
4	Right	70–80	249	61	Overall stable since June 1997	0	93	31
5	Left	80–90	300	90	Reduced from >90% in August 1996	0	97	30
6	Right	60–70	191	37	Progression from 50–60% in November 1996	0	81	20
7	Left	90–99	506	219	Stable since July 1998	0	51	24
8	Left	90–99	392	134	Stable since September 1996	<50	120	43
9	Right	90–99	343	93	Stable since November 1997	80–90	310	54
10	Left	70–80	243	58	Stable since February 1999	60–70	186	53

†*PSV, peak systolic velocity; EDV, end diastolic velocity; ICA, internal carotid artery*.

* *Subject two was recruited to the ASED study from the Box Hill Hospital using a left ICA PSV of ≥ 150 centimetres/second, other criteria derived from Australasian Society for Ultrasound Medicine and the mid-point of the reported stenosis range (see main text)*.

**Table 2:** Demographics, other risk factors and medication taken during 24-h TCD monitoring^†^.

**Patient No**.	**1**	**2**	**3**	**4**	**5**	**6**	**7**	**8**	**9**	**10**	**Mean/total**
Age (years)	81	85	68	76	70	90	66	83	84	85	Mean 79
Sex	M	M	M	M	F	M	M	M	M	F	80% male
Hypertensive	Yes	No	Yes	No	Yes	Yes	Yes	Yes	Yes	Yes	80%
Hypercholesterolaemic	Yes	Yes	No	Yes	Yes	Yes	Yes	Yes	Yes	Yes	90%
Current smoker	No	No	No	No	No	No	No	No	Yes	No	10%
Prior smoker	Yes	No	Yes	No	Yes	Yes	Yes	Yes	No	No	60%
Diabetes mellitus	No	No	No	No	No	No	No	No	No	No	0%
Ischaemic heart disease	No	Yes	No	No	No	Yes	Yes	Yes	Yes	No	50%
Other prior stroke/TIA	No	Yes	Yes	No	No	Yes	Yes	No	No	No	40%
Peripheral arterial disease	Yes	No	Yes	No	Yes	No	No	Yes	Yes	No	50%
Prior confirmed sleep apnoea	No	No	Yes	No	Yes	No	No	No	Yes	No	30%
On ≥1 antiplatelet drug	Yes	Yes	Yes	Yes	Yes	Yes	Yes	Yes	Yes	Yes	100%
On statin therapy	Yes	No	Yes	No	Yes	No	No	Yes	Yes	Yes	60%
On ≥1 anti-hypertensive drug	Yes	No	Yes	Yes	Yes	Yes	Yes	Yes	Yes	Yes	90%

† *As recorded during follow-up in the ASED Study in the months prior to 24-h recording. Any prior stroke or TIA did not involve the study carotid artery territory. TCD, transcranial Doppler; ASED, Asymptomatic stenosis embolus detection*.

### General Embolic Signal Detection Results

#### Study Arteries

All 10 subjects, each with one “study” artery with 60–99% asymptomatic carotid stenosis, were successfully monitored for 24 h. After taking into account monitoring breaks and periods of inadequate signal, there was a total of 177.9 h of effective monitoring from the 10 study arteries (range 12.0–21.3 and mean 17.8 h/study artery). A total of 56 ES were detected from five of these 10 study arteries (Tables 3, 4). No ES were detected for the remaining 5 study arteries. When the 24-h day was divided into four 6-h intervals and combining results from all 10 study arteries, the total in effective monitoring hours ranged from 41 to 48/interval, being lowest for 0600-midday (Table 4). When the 24-h day was divided into six 4-h intervals and combining results from all 10 study arteries, the total in effective monitoring hours ranged from 25 to 34/interval, being lowest for 0800-midday (Table 5).

**Table 3:** Embolic signal counts and effective monitoring time for study arteries according to 6-hourly intervals from midnight^†^.

**Patient/study artery No**.		**Midnight-0600**		**0600-midday**		**Midday-1800**		**1800-midnight**		**Total ES**	**Total Time**
		**ES**	**Time**	**ES**	**Time**	**ES**	**Time**	**ES**	**Time**		
1		**1**	334	**3**	278	**0**	92	**0**	200	**4**	904 **(14.9 h)**
2		**0**	290	**1**	222	**0**	267	**0**	226	**1**	1005 **(16.8 h)**
3		**0**	355	**0**	238	**3**	302	**0**	328	**3**	1223 **(20.4 h)**
4		**0**	345	**0**	346	**0**	317	**0**	270	**0**	1278 **(21.3 h)**
5		**0**	351	**0**	195	**0**	290	**0**	270	**0**	1106 (**18.4 h**)
6		**3**	396	**7**	192	6	293	4	330	**20**	1211 (**20.2 h)**
7		**3**	264	**15**	263	**10**	305	**0**	230	**28**	1062 **(17.7 h)**
8		**0**	182	**0**	156	**0**	250	**0**	336	**0**	924 (**15.4 h)**
9		**0**	30	**0**	272	**0**	215	**0**	200	**0**	717 (**12.0 h)**
10		**0**	325	**0**	289	**0**	280	**0**	349	**0**	1243 (**20.7 h)**
Totals/ time of day	ES detected	**7**		**26**		**19**		**4**		**56**	
	Effective monitoring time	2872 min = **47.87 h**		2451 min = **40.85 h**		2611 min = **43.52 h**		2739 min = **45.65 h**		10673 min = **177.88 h**	
Average ES/effective monitoring hour by time of day		**0.15**		**0.64 (PEAK)**		**0.44**		**0.09 (TROUGH)**		**0.31**	

† *Time is in minutes unless otherwise specified. ES, embolic signal; ES, embolic signal*.

**Table 4:** Embolic signal counts and effective monitoring time for study arteries according to 4-hourly intervals from midnight^†^.

**Patient/study artery No**.		**Midnight-0400**		**0400–0800**		**0800-Midday**		**Midday-1600**		**1600–2000**		**2000–2400**		**Total ES**	**Total time**
		**ES**	**Time**	**ES**	**Time**	**ES**	**Time**	**ES**	**Time**	**ES**	**Time**	**ES**	**Time**		
1		**0**	240	**1**	207	**3**	165	**0**	52	**0**	75	**0**	165	**4**	904 **(15.1 h)**
2		**0**	220	**0**	103	**1**	189	**0**	186	**0**	148	**0**	159	**1**	1005 **(16.8 h)**
3		**0**	235	**0**	233	**0**	125	**2**	207	**1**	215	**0**	208	**3**	1223 **(20.4 h)**
4		**0**	240	**0**	225	**0**	226	**0**	195	**0**	202	**0**	190	**0**	1278 **(21.3 h)**
5		**0**	240	**0**	191	**0**	115	**0**	185	**0**	219	**0**	156	**0**	1106 **(18.4 h)**
6		**3**	276	**5**	200	**2**	112	**3**	198	**3**	185	**4**	240	**20**	1211 **(20.2 h)**
7		**1**	204	**7**	165	**10**	158	**9**	191	**1**	139	**0**	205	**28**	1062 **(17.7 h)**
8		**0**	182	**0**	0	**0**	156	**0**	137	**0**	228	**0**	221	**0**	924 **(15.4 h)**
9		**0**	0	**0**	223	**0**	79	**0**	130	**0**	190	**0**	95	**0**	717 **(12.0 h)**
10		**0**	225	**0**	218	**0**	171	**0**	190	**0**	214	**0**	225	**0**	1243 **(20.7 h)**
Totals/ time of day	ES detected	**4**		**13**		**16**		**14**		**5**		**4**		**56**	
	Effective monitoring time	2062 min = **34.37 h**		1765 min = **29.42 h**		1496 min = **24.93 h**		1671 min = **27.85 h**		1815 min = **30.25 h**		1864 min = **31.07 h**		10673 min = **177.88 h**	
Average ES/effective monitoring hour by time of day		**0.12 (TROUGH)**		**0.44**		**0.64 (PEAK)**		**0.50**		**0.17**		**0.13**		**0.31**	

† *Time is in minutes unless otherwise specified. ES, embolic signal*.

**Table 5:** Embolic signal detection according to 6-monthly, 60-min studies vs. the 24-h study^†^.

**Patient/study artery no**.	**6-monthly, 60-min studies BEFORE the 24- h study**		**ES/artery on 24-h study**	**6-monthly, 60-min studies AFTER the 24- h study**	
	**No. of studies**	**Number and timing of all ES relative to the 24-h study**		**No. of studies**	**Number and timing of all ES relative to the 24-h study**
1	4	**2 ES 349 days before and 1 ES 167 days before**	**4**	2	0
2	2	0	**2**	3	**1 ES 484 days after**
3	3	0	**3**	3	**1ES 486 days after**
4	7	**1 ES 690 days before**	0	2	0
5	8	**1 ES 399 days before**	0	1	0
6	5	**1 ES 853 days before**	**20**	2	0
7	5	**1 ES 250 days before**	**28**	1	**7 ES 161 days after**
8	9	0	0	1	0
9	6	0	0	2	**1 ES 187 days after**
10	3	0	0	2	STA artefact 201 days after

† *ES, embolic signal; STA, superficial temporal artery*.

The total ES detected over the 24 h ranged from 0 to 28/study artery (Tables 4, 5). The average hourly ES detection rate ranged from 0 to 1.6/study artery. The average intensity of each of the 56 ES was 6.79 dB (range 5.5–16 dB) above the background MCA blood flow signal. Detection of clusters of emboli occurred. The best example occurred in the case with the largest ES total in whom 28 ES were detected in 17.7 total effective monitoring hours. However, 16 of these ES were detected in a 3.5 h block starting in the late-morning session (between 11.00 and 14.30). The longest ES-free period during for this artery was 11 h (16.20–03.20).

The yield of detecting ≥1 ES using 24-h and ASED Study 6-monthly, 60-min TCD monitoring was compared (Table 5). ES detection results were consistent for 7 of the 10 study arteries in that ES were detected (5 cases) or never detected (2 cases) using both 24-h and 6-monthly 60-min monitoring. However, in one case (subject 10] where ES were never detected, one of the 6-monthly, 60-min studies was uninterpretable due to frequently recurrent high-intensity signals looking and sounding much like ES but attributed to superficial temporal artery (STA) pulsation (1). In the remaining 3 cases, results were inconsistent with no ES detected during the 24-h study and, yet, ES were detected during the prior or subsequent 6-monthly, 60-min studies.

#### Contralateral Arteries

Two of the 10 subjects had bilateral 60–99% asymptomatic ICA stenosis (Table 1), while two had mild contralateral ICA stenosis (<50%), one had a contralateral ICA occlusion and the remaining five had contralateral ICA non-stenosing plaque (one after prior CEA). Twenty-four-hour monitoring was unsuccessful in two of these contralateral “control” arteries. In subject 10, with contralateral 60–70% ICA stenosis, the ipsilateral MCA TCD signal was uninterpretable due to recurring frequent high intensity signals throughout almost the entire study which (as above) was attributed to STA pulsation (1). Twenty-four-hour monitoring from this contralateral artery was excluded because ES could not be differentiated from this artefact. Further, the contralateral MCA of subject six (with ipsilateral non-stenosing ICA plaque) was not monitored due to equipment failure.

Considering all eight contralateral monitored arteries, there was a total of 134.7 effective monitoring hours (mean 16.8 and range of 10.1–20.7 h/artery). Depending on the monitoring interval used, the lowest total in effective monitoring hours was midnight-0400 (11.3 h) or midnight-0600 (16.5 h) when the ipsilateral TCD probe was often removed to allow subjects to sleep. Totals for other monitoring intervals varied between 16.6 and 30.5 h (using 4-hourly intervals) or 35.3–41.8 h (using 6-hourly intervals). No ES were detected from the MCAs ipsilateral to the eight monitored contralateral carotid arteries.

### Embolic Signal Detection Rates in Study Arteries With Advanced Asymptomatic Carotid Stenosis According to Time of Day

#### Six-Hourly Intervals From Midnight (Table 3 and Figure 1A)

The mean ES detection rate was highest 0600-midday (0.64/h) followed by midday-1800 (0.44/h). Mean ES detection rates were lowest 1800-midnight (0.09/h) before beginning to rise again midnight-0600 (0.15/h). Peak ES rates were between 0600-midday in four of the five ES-positive arteries, with a peak straddling this time in the remaining case. The difference in rate extremes, between 0600-midday and 1800-midnight, was statistically significant, as indicated by the incidence rate ratio of 7.26 (95% CI 2.52–28.64, *P* ≤ 0.001).

**Figure 1:**
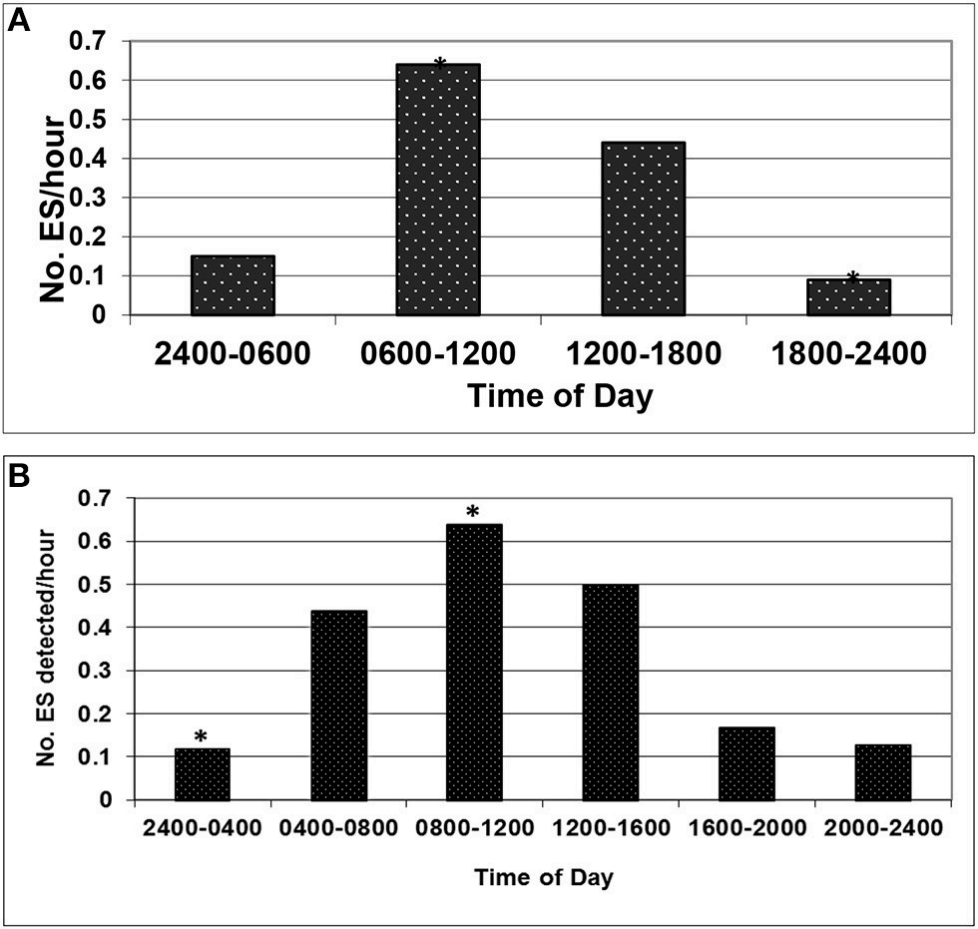
**(A)** The difference in rate extremes, between 0600-midday^*^ and 1800-midnight^*^, was statistically significant: incidence rate ratio 7.26 (95% CI 2.52–28.64, *P* ≤ 0.001). **(B)** The difference in rate extremes, between 0800-midday^*^ and midnight-0400^*^, was statistically significant: incidence rate ratio 5.52 (95% CI 1.78–22.67, *P* = 0.001).

### Four-Hourly Intervals From Midnight (Table 4 and Figure 1B)

The mean ES detection rate was highest 0800-midday (0.64/h) followed by midday-1600 (0.50/h). Mean ES detection rates were lowest midnight-0400 (0.12/h), very similar to the rate from 2000-midnight (0.13/h). Peak ES rates were between 0800-midday in three of the five ES-positive arteries, with peaks straddling this time in the remaining cases. The difference in rate extremes, between 0800-midday and midnight-0400, was statistically significant, as indicated by the incidence rate ratio of 5.52 (95% CI 1.78–22.67, *P* = 0.001).

### Sleep Study Results

The estimated pre-sleep study probability of having OSA was low in 3 cases. In one of these (subject one) OSA was diagnosed on the sleep study, while one (subject two) had apneas without desaturation and the other (subject four) had no evidence of OSA. The estimated pre-sleep study probability having significant OSA was high in the remaining seven subjects (including three with previous sleep study-confirmed OSA). OSA was demonstrated in six of these seven subjects (subjects 3, 5, 6, 8, 9, 10), while the remaining subject (subject seven) had rapid eye movement (REM) sleep disordered breathing, in which cyclical fluctuations in the respiratory signals did not meet the criteria to be scored as apneas or hypopneas. Five subjects (2, 3, 6, 9, and 10) were newly or re-prescribed CPAP following the sleep study. Subject five was CPAP intolerant.

### Outcome Events of Interest During Follow-Up

The 10 subjects were follow-up clinically as part of the ASED Study for an average of 502 days after the 24-h TCD study (range 213–638 days). Two subjects had a stroke during this time. Subject two had ipsilateral TIAs then an ischaemic stroke 623 days after the 24-h study. Each episode consisted of mild right upper limb paresis and parasthesiae. Eight days after the stroke he had an endarterectomy for a macroscopically ulcerated, haemorrhagic and degenerate left ICA plaque causing 70–79% stenosis (on preoperative duplex and at surgery) and was discharged home. Subject seven had a severe ipsilateral ischaemic stroke with right hemiparesis, dysphasia, dysphagia and seizures 213 days after the 24-h study in association with a new left ICA occlusion and was discharged to a nursing home. There were no other strokes or ipsilateral carotid procedures and no TIAs, myocardial infarctions or deaths during follow-up after 24-h monitoring.

## Discussion

We tested the validity ES detection in persons with advanced asymptomatic carotid stenosis using different TCD monitoring durations, frequencies and times of day. This process is necessary for accurately risk stratifying individuals and appropriate treatment decisions. Strengths of our work include our meticulous study methods and many hours of effective monitoring (1,407 h from 60-min TCD studies and 178 h from 24-h monitoring) despite the very labour-intensive nature of TCD embolus detection in this population. Notable temporal variability in ES detection has been reported associated with asymptomatic and symptomatic carotid stenosis using repeated 40–60 min or relatively prolonged (5 h) continuous TCD monitoring (1–3, 30, 31). Our present findings build on this previous work. In addition, a trend towards circadian variation in TCD ES detection, with a morning peak, was reported in a cohort of just seven symptomatic persons with potential carotid, aortic or cardiac embolic sources (4). However, our study is the first to demonstrate a circadian variation in ES detection related to asymptomatic carotid stenosis.

We studied individuals with duplex determined 60–99% asymptomatic carotid stenosis and found a peak average ES detection rate of 0.64/h in the 4–6 h before midday. This was seven or five times higher than trough ES detection rates (depending, respectively, on 6- or 4-hourly monitoring intervals). Our finding is consistent with previous observations of higher ES detection rates following CEA performed in the morning compared to the afternoon (5). They are also consistent with the well-documented late-morning peak in the incidence of symptomatic arterial and venous disease complications (6–13) and factors implicated in the pathogenesis of such events (14–18, 20).

The circadian variation in ES detection associated with advanced asymptomatic carotid stenosis was shown with only 10 subjects. This implies strong biological effect. Our results were not simply due an outlier, such as subject seven with the highest total ES count. Peak ES rates were between 0600-midday or 0800-midday in most ES-positive arteries, with peak rates straddling these times in the remaining cases. In the very least, our study should stimulate others to consider the influence of time of day on ES detection results. Circadian regulation of biological function occurs in many ways (32, 33). Evidently this provides survival advantage by supporting peak human activity in the morning with progressive “winding down” from midday through the evening until rising again just prior to, and during, the usual waking hours. However, peak activity carries a higher risk of thrombotic events. Understanding this vulnerability provides opportunity for improving preventative strategies (32, 33).

Of interest, peak activity (or incidence) of some factors associated with symptomatic vascular events occurs just before or just following late morning, giving clues to pathogenesis. For example, blood plasminogen activator inhibitor levels peak and tissue-type plasminogen activator (t-PA) levels trough at about 0300 (19) indicating several hours lag between peak clot formation and symptomatic events. Peak asymptomatic “ST-segment elevation” in patients with “variant” or more typical angina occurs at 0400–0700, implying lag between asymptomatic and symptomatic states (34, 35). The frequency of ventricular arrhythmias is highest in the 1–6 h before and/or 6 h after midday (36–41). This tendency to overlap and/or follow the peak incidence of thrombotic events is possibly because reperfusion (when ST segments are falling) is an important cause of ventricular arrhythmias (34).

Our results demonstrate that *time of day* and *monitoring duration* influence the ES-detection yield. During 24-h TCD monitoring, 1–23 h of monitoring was insufficient to identify the five ES-positive arteries due to gaps which were more likely overnight. We have shown that office hours provide the best yield with respect to ES detection in relation to advanced asymptomatic carotid stenosis. These hours are also best due to staff availability and avoid overnight monitoring which may be uncomfortable. It is also known that ES detection yield increases with *monitoring frequency*. For example, at completion of ASED Study data collection in 2002, there were 1,000 technically adequate hours of 6-monthly, 60-min monitoring from 202 subjects with 231 study arteries with 60–99% asymptomatic carotid stenosis monitored at least once and on average 4.3 times. Using these results, 60 arteries (26%) were ES-positive with ≥1 ES detected at least once (1). Further, 23 subjects had eight-ten 6-monthly, 60-min TCD studies. Using these results, the proportion with ≥1 ES detected rose from 12% after one 60-min study to 41% by 8–10 studies (1).

In the ASED study ES detection variability was also tested, when subjects consented, by performing “additional” 60-min TCD studies immediately or up to 6 months after scheduled 60-min studies (unpublished data). These additional studies were scheduled blinded to ES detection results. There were 407 technically adequate “additional” 60-min studies from 202 of the ASED study arteries. This made a total of 1,407 ASED Study effective monitoring hours (310 total ES detected in 68/231 study arteries; median 0, mean 1.3, and range 0–82 ES/monitoring hour/study artery, including 58 “short” studies of 15–59 min duration). Using all 1,407 monitoring hours, each of the 231 study arteries was monitored on average six times. Using these results, 68 arteries (29%) were ES-positive. By contrast, and again using all 1,407 monitoring hours, 13 subjects with 13 study arteries were monitored for 60 min 12–15 times. Using these results, eight arteries (62%) were ES-positive. This high ES prevalence and other work showing average annual ipsilateral stroke rates of ≥1% in relation to advanced asymptomatic carotid stenosis indicate that detecting ≥1 ES lacks sufficient specificity to justify carotid procedures aimed to prevent stroke in asymptomatic individuals (42–44).

ES-detection yield depends *on time of day* and the *monitoring frequency* and *duration*. Five of the 10 subjects were identified as ES-positive with 24-h TCD monitoring, including two who were not identified as ES-positive using 6-monthly, 60-min monitoring until after the 24-h study and three who were previously ES-positive using 6-monthly, 60-min monitoring. For each of the five arteries identified as ES-positive with 24-h monitoring, on four-eight different occasions, a 6-monthly, 60-min TCD study did not identify them as ES-positive. Further, 3 of the 10 arteries were ES-negative with the 24-h study and yet were ES-positive considering their eight-nine 6-monthly, 60-min studies. Therefore, in our study of 10 persons with advanced asymptomatic carotid stenosis, 24-h monitoring sometimes identified ES-positive arteries sooner than 6-monthly 60-min monitoring and it increased the ES-detection yield depending on how many 6-monthly 60-min studies were done and when they were done. However, overall, an average of 7.1 6-monthly, 60-min TCD studies/artery identified more arteries as ES-positive than 24 h of monitoring spaced over two consecutive days.

Our observations underscore that many hours of monitoring are required to properly distinguish embolising and non-embolising advanced (60–99%) asymptomatic carotid stenosis because of low overall ES rates and temporal variability, including due to circadian influences. This variability is likely related to pathological changes in the carotid artery, or elsewhere proximal to the monitored MCA portion. Our results also indicate that any ipsilateral microembolism is common in relation to asymptomatic carotid stenosis with ≥1 ES found in ≥62% of subjects. In the great majority, these are of no direct or immediate clinical consequence (1). Our results are consistent with a study of 12 symptomatic patients with ipsilateral carotid stenosis by Mackinnon et al. in which 7 (58%) were ES-positive after 1 h of monitoring and 11 (92%) by 5 h (31). One to 4.5 h of monitoring was usually insufficient to identify embolisation in symptomatic individuals due to ES gaps. Compared to asymptomatic individuals, symptomatic persons with ipsilateral carotid stenosis, overall, are about 3–4 times more likely to have ES detected and ES detection rates are about 20–55 times higher (45, 46).

Other known factors (apart from time of day, frequency and duration of monitoring and symptomatic status) that increase the likelihood of detecting ES with TCD are shorter time since stroke or TIA, presence of ipsilateral carotid stenosis (compared to no stenosis) and increasing carotid stenosis severity (46). Factors associated with a reduced likelihood of detecting ES are presence of critical (>90–95%) carotid stenosis and use of antiplatelet or statin therapy or CEA in symptomatic persons (46–49). Of note CEA does not always abolish ES, indicating the proximal ICA is an ongoing source of embolism and/or the presence of other sources (50).

TCD ES detection has been shown to identify symptomatic patients with carotid stenosis (51) or those undergoing CEA, at higher risk of stroke or TIA (28). These patients have relatively high rates of both ES and events. However, ES detection has not been shown to identify persons with asymptomatic carotid stenosis at higher stroke risk, or likely to benefit from a carotid procedure, despite what some guidelines state (52). Of the three studies examining the association between ES detection and subsequent stroke risk in asymptomatic subjects with advanced carotid stenosis, “ASED” (1), “ACES” (2), and a Canadian study (3), two were uninterpretable (and likely underpowered) because nearly half of the eight (2) and 10 (3) ipsilateral strokes, respectively, that occurred during follow-up (and used in analyses) were in those who first had an ipsilateral TIA during follow-up. Therefore, these were actually studies of ES detection in mixed samples of asymptomatic and symptomatic patients with carotid stenosis. The third study was negative (1). Further, if ES detection is one day shown to identify those at significantly higher risk of ipsilateral stroke despite current optimal medical intervention, randomised trials will then be required to determine any additional procedural benefit.

Much larger sample sizes are required to further explore the association between ES detection and stroke risk in relation to asymptomatic carotid stenosis, including the importance of different embolic rates, recurrent ES detection and better medical intervention for arterial disease (risk factor identification, lifestyle coaching and medication). This is indicated by very low overall rates of ES and strokes in previous studies (1–3) and ongoing improvements in medical intervention since they were performed (42, 43). For example, medical intervention in the ASED study could have been improved by more direct investigator intervention to reduce subject blood pressure and lipid levels. However, it was the ASED and similar studies which produced a new appreciation of the total stroke prevention effectiveness of reducing all vascular disease risk factors (42, 43, 53).

Unfortunately, two subjects selected for 24-h TCD later had ipsilateral strokes. Neither was receiving statin therapy, likely to be given in current practice. Both were on antiplatelet therapy because of prior symptomatic arterial disease. In addition, both subjects were ES-positive on 24-h monitoring, with the lowest and highest total 24-h ES counts and both had ES subsequently detected. However, the 24-h study involved preselected ES-positive individuals and was not designed to test the stroke risk stratification power of ES detection. Moreover, as above, the available evidence from the ASED and other studies indicates that detecting ≥1 ES in such individuals lacks sufficient specificity to identify those likely to have a stroke or benefit from carotid endarterectomy or stenting. This is because detecting ≥1 ES is expected in ≥62% of such individuals (if monitored long or often enough), whereas the average annual stroke rate associated with advanced asymptomatic carotid stenosis is now approximately only 0.5–1.0% or less (44). New and larger studies are required to test the value of detecting more than one ES. However, It is expected from studies of ES detection after CEA, that higher ES rates will be associated with higher stroke risk (28).

In addition, larger studies could check for a secondary afternoon peak in ES rates (4), add certainty about the usual timing of the overall trough in ES detection in relation to midnight and test for the effect of different diagnostic criteria for ES, such as signal intensity. We chose a 6 dB intensity threshold based on preliminary investigations (23, 26, 27). This facilitated differentiation of ES from artefact and normal background signal variations. While lower ES intensity thresholds may improve ES detection sensitivity, they are also likely to reduce specificity (27). Further, lower intensity thresholds may not improve overall accuracy or be required for clinical significance. Larger and differently designed studies would be required to test for seasonal variation in ES detection.

Unfortunately, further study of the asymptomatic population is particularly problematic because labour-intensive, manual methods of ES detection are required given the relatively infrequent and low intensity ES compared to other populations (1). Reliable automated methods of detecting ES are not available for this population. Moreover, ES detection is likely to be more useful in stratifying overall risk of any arterial disease complication in asymptomatic individuals or better selecting symptomatic patients for CEA (44). Although a portable ES detection system has been described (31), we do not think using such a system would have impacted significantly on our results because artefacts still occur and the breaks would still have been required for meals and overnight with subjects sleeping on one side.

OSA was diagnosed in eight of our 10 subjects with advanced asymptomatic carotid stenosis, including one who had no clinical features to suggest its presence. OSA is common in individuals with arterial disease, is often unrecognised and is a risk factor for arterial disease complications (54, 55). While CPAP improves secondary symptoms of OSA and blood pressure control, so far studies have not shown that it reduces arterial complications (54). Our sample was too small to test the prevalence of ES in those with or without OSA. However, both conditions affected most of our subjects. The late morning peak in ES detection rates and symptomatic thrombotic events argues against OSA directly or immediately precipitating most of these events. More likely OSA contributes to a multifactorial dynamic process from which symptomatic complications are the “tip of the iceberg” result.

## Conclusion

Using a total of 1,585 h of effective hours of TCD monitoring, we have demonstrated that ES detection yields in relation to advanced asymptomatic carotid stenosis are significantly influenced by the time of day and the duration and frequency of monitoring. We identified peak ES detection rates in the 4–6 h prior to midday, consistent with peak incidence in symptomatic vascular complications. In addition, we have demonstrated, in several ways, that 1 h (and sometimes many more hours) of TCD monitoring is insufficient to reliably distinguish embolising from non-embolising carotid arteries. Further, our work has shown that that ≥62% of persons with advanced asymptomatic carotid stenosis will have ≥1 ES detected if they are monitored often enough. Therefore, detecting ≥1 ES in this population lacks sufficient specificity to identify the small proportion of asymptomatic persons now likely to benefit from a carotid procedure aimed to prevent stroke (42–44). However, TCD ES detection may play a valuable role in measures to reduce the risk of stroke and other vascular complications (33). Although our study was not designed or powered to test the influence other factors (such as medical treatments, age, sex, or plaque characteristics) on ES detection in relation to asymptomatic carotid stenosis, we have provided important insights for optimizing such studies.

## Author's Note

This work was largely completed at the National Stroke Research Institute, Austin Health, Melbourne, Australia (now part of the Florey Institute of Neuroscience and Mental Health, Melbourne, Australia), in association with the University of Melbourne, Melbourne, Australia.

## Author Contributions

AA: concept, design, ethics approvals, grant funding procurement, patient recruitment and baseline assessments, data analysis, manuscript drafting, editing, approval, and submission; JM: patient recruitment and baseline assessments, all 24-h TCD monitoring and patient supervision, data analysis, manuscript editing, and approval; DP: statistical analyses, manuscript drafting, editing, and approval; AJ: data analysis, manuscript editing, and approval; CW: sleep studies and sleep study analyses, manuscript editing, and approval; EF: literature review, manuscript editing, and approval; BC: overall responsibility for the study, manuscript drafting, editing, and approval.

### Conflict of Interest Statement

AA and EF would like to acknowledge their membership in the Faculty Advocating Thoughtful and Collaborative Carotid Artery Treatments (FACTCATS at FACTCATS.org). All authors declare that the research was conducted in the absence of any commercial or financial relationships that could be construed as a potential conflict of interest.
