# Supplementary material for: Asymptomatic Carotid Stenosis Is Associated With Circadian and Other Variability in Embolus Detection
Source: Front Neurol. 2019 Apr 16;10:322. doi: 10.3389/fneur.2019.00322 (PMC6476949; doi:10.3389/fneur.2019.00322)
Supplement: Supplementary file 1 [file Data_Sheet_1.docx]

**Asymptomatic Carotid Stenosis is Associated with Circadian and Other Variability in Embolus Detection**

**Anne L Abbott ^1^, Julia Merican ^2^, Dora C Pearce ^3 4^**, **Ana Juric,**

**Christopher Worsnop ^5^, Emma Foster ^6 7^, Brian Chambers ^8 9 10^**

**Supplementary Material Only**

**Supplementary Table 1. Method of measuring carotid stenosis in the ASED Study at the Austin Hospital***

| 1. **Zwiebel, 1994 (1)** | |
| --- | --- |
| **Diameter stenosis (%)** | **PSV centimeters/second (cm/sec)** |
| 50 | 120-150 |
| 60 | 150-170 |
| 60-70 | 170-220 |
| 70 | 170-220 |
| 70-80 | 250-290 |
| 80 | 290-300 |
| 90 | >300 |

| 1. **Bluth et al, 1998 (2)** | | | | |
| --- | --- | --- | --- | --- |
| **Diameter Stenosis (%)** | **PSV cm/sec** | **PEDV cm/sec** | **Ratio PSV ICA/CCA** | **Ratio PEDV ICA/CCA** |
| 0 | <110 | <40 | <1.8 | <2.6 |
| 1-39 | <110 | <40 | <1.8 | <2.6 |
| 40-59 | <130 | <40 | <1.8 | <2.6 |
| 60-79 | >130 | >40 | >1.8 | >2.6 |
| 80-99 | >250 | >100 | >3.7 | >5.5 |

*PSV= Peak systolic velocity; PEDV= Peak end diastolic velocity; ICA= internal carotid artery; CCA= common carotid artery; cm/sec= centimeters per second

Supplementary Table 2. Method of measuring carotid stenosis in the ASED Study at the Box Hill and John Hunter Hospitals (3)*

| Stenosis grade | Ultrasound criteria | |
| --- | --- | --- |
| 0 | Normal waveform and image | |
| <15% | PSV < 125cm/sec | Deceleration spectral broadening |
| 16-49% | PSV < 125cm/sec | Pansystolic spectral broadening |
| 50-69% | PSV > 125cm/sec and EDV < 110cm/sec or ICA/CCA >2 | Pansystolic spectral broadening |
| 70-79% | PSV > 270 cm/sec or  EDV >110cm/sec or  ICA/CCA > 4 | Pansystolic spectral broadening |
| 80-99% | As above plus EDV > 140 cm/sec | Pansystolic spectral broadening |
| Occluded | No flow, terminal thump | |

*ICA = Internal carotid artery; CCA = Common carotid artery; PSV = Peak systolic velocity; EDV = End diastolic velocity; ICA/CCA = Ratio of ICA PSV to CCA PSV; cm/sec = centimeters per second

References for Supplementary Tables:

1. Zweibel W. Unpublished presentation. *Australian Society of Ultrasound Medicine* 1994

1. Bluth EI, Stavros AT, Marich KW, Wetzner SM, Aufrichtig D, Baker JD. Carotid duplex sonography: A multicenter recommendation for standardized imaging and Doppler criteria. *Radiographics* 1988;8:487-506
2. Australasian Society for Ultrasound Medicine 1998 conference presentation and subsequently published by the Australasian Society for Ultrasound in Medicine - Guidelines, Policies and Statements D14. Accessible at:  [https://www.asum.com.au/files/public/SoP/Current/Vascular/Duplex-Ultrasound-Extracranial-Carotid-Artery-Disease-D14.pdf.](Https://www.Asum.Com.Au/files/public/sop/current/vascular/duplex-ultrasound-extracranial-carotid-artery-disease-d14.Pdf) (last accessed 30^th^ January 2019)
